# The complete chloroplast genome sequence of *Malus halliana* (Rosaceae), an important ornamental plant

**DOI:** 10.1080/23802359.2020.1768936

**Published:** 2020-05-27

**Authors:** Shuai Wang, Jing Yang, Bin Zhang, Fude Shang, Hongwei Wang

**Affiliations:** a College of Life Science, Henan Agricultural University, Zhengzhou, China; b College of Forestry, Henan Agricultural University, Zhengzhou, China; c College of Plant Protection, Henan Agricultural University, Zhengzhou, China

**Keywords:** *Malus halliana*, chloroplast genome, Rosaceae, phylogenetic analysis

## Abstract

*Malus halliana* is an important ornamental plant resource. Herein, we determined the complete chloroplast (cp) genome sequence of *M. halliana* using Illumina sequencing data. The whole cp genome is 160,089 bp in size, consisting of a pair of inverted repeats (IR 52,706 bp), a large single-copy region (LSC 88,189 bp), and a small single-copy region (SSC 19,194 bp). The plastid genome contains 129 genes, 84 protein-coding genes, 37 tRNA genes, and eight rRNA genes. In addition, a maximum-likelihood phylogenetic analysis demonstrated that *M. halliana* was most closely related to *Malus hupehensis*. The complete plastome sequence will provide useful genetic information for phylogenetic studies, the resolution of taxonomic discrepancies, and molecular breeding.

*Malus halliana* Koehne is a woody ornamental species of *Malus* in Rosaceae that is endemic to China (Sun et al. 2011). Its flower pedicels are slender and drooping, the flowers are brightly colored, and many flowers are clustered in umbrella-shaped inflorescences; therefore, this species has high ornamental value as a famous horticultural resource (Sun et al. 2011; Yin et al. 2017). However, the taxonomy and relationships of the genus *Malus* are complex, and the systematic position of many species is unclear, including *M. halliana* (Robinson et al. 2001). Therefore, we reported the complete chloroplast genome (cp) of *M. halliana* based on Illumina pair-end sequencing data, probed its systematic position in *Malus*.

The leaf sample was collected from Bishagang Park (34°44′59″N, 113°37′27″E, alt. 107 m), Zhengzhou City, China. The specimen was deposited in the Herbarium of Henan Agricultural University (specimen code WS20190403). The E.Z.N.A.^®^ HP Plant DNA Kit (OMEGA) was used to extract the whole genomic DNA of *M. halliana* (Abdulamir et al. 2010). The concentration of DNA was checked by using a TBS380 system (Picogreen). The library was sequenced via the Illumina HiSeq double terminal sequencing method.

The raw data were filtered to remove reads with low sequencing quality values and duplicated reads to obtain high-quality clean data. We used the chloroplast-like reads to assemble sequences by using NOVOPlasty-v2.7.2 (Dierckxsens et al. 2017). GapCloser-1.12 was used to extend sequences and fill gaps (Acemel et al. 2016). The physical map of the new chloroplast genome was generated using OGDRAW (Lohse et al. 2013). Finally, the validated complete cp genome sequence was submitted to GenBank (accession number: MT246302).

The complete cp genome of *M. halliana* is 160,089 bp in length, containing a large single-copy (LSC) region of 88,189 bp, a small single-copy (SSC) region of 19,193 bp, and two inverted repeat (IR) regions of 26,352 bp. The new sequence contains a total of 129 genes, including 84 protein-coding genes, 37 tRNA genes, and eight rRNA genes. The overall GC content of the whole plastome is 36.55%, whereas the corresponding values of the LSC, SSC, and IR regions are 34.21%, 30.37%, and 42.70%, respectively.

The maximum-likelihood phylogenetic tree was reconstructed based on the entire chloroplast genome sequences of *M. halliana* and ten other *Malus* species using *Cydonia oblonga* as the outgroup species. The results indicated that *M. halliana* was closely related to *Malus hupehensis* with 100% bootstrap support (Figure 1). The complete plastome of *M. halliana* will provide useful genetic information for phylogenetic studies, the resolution of taxonomic discrepancies, and molecular breeding.

**Figure 1.**
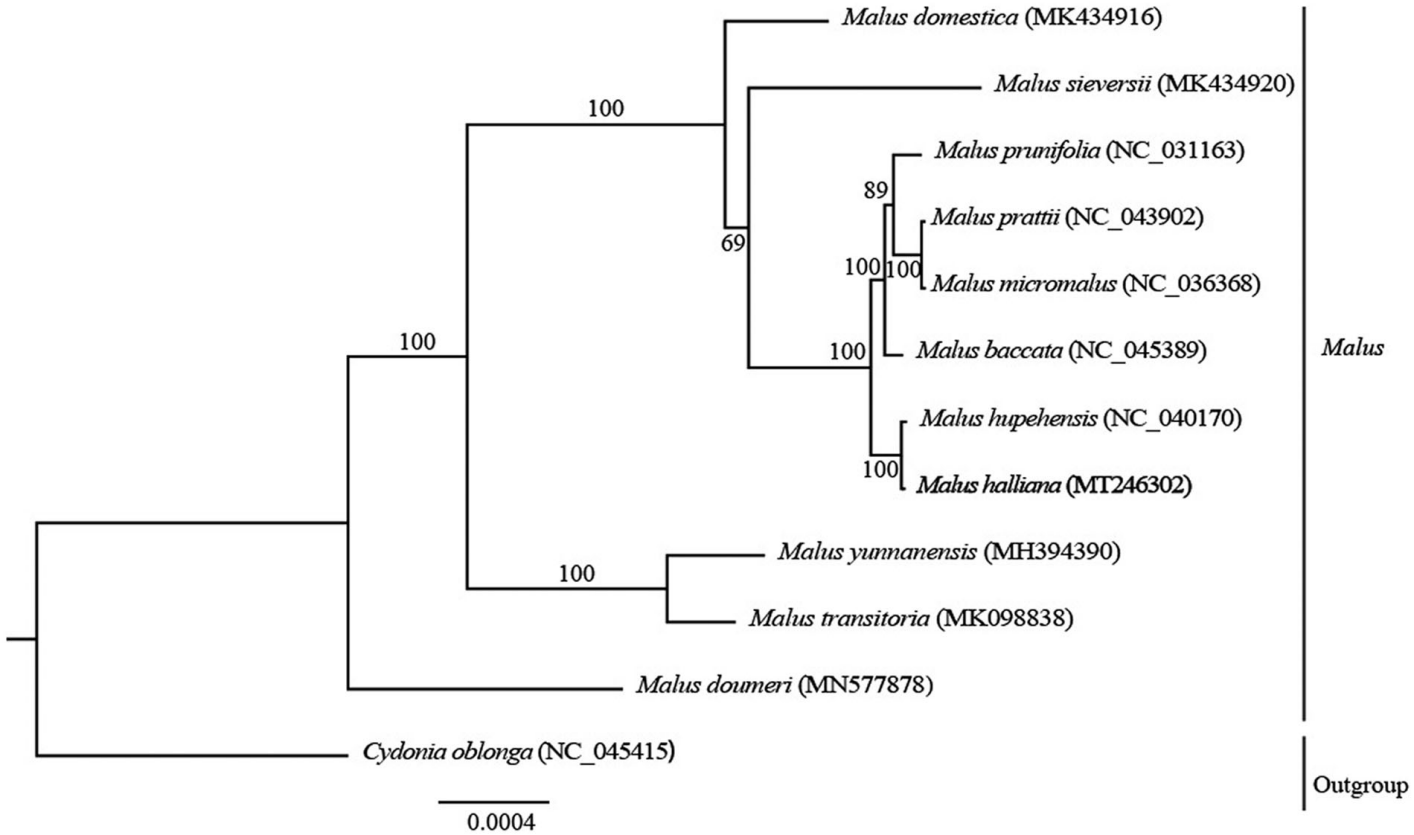
A maximum-likelihood phylogenetic tree was constructed including 12 species based on entire complete chloroplast genomes. Bootstrap support values are shown above or under the branches. The new complete cp genomes obtained in this study are shown in bold.

## Data Availability

The data that support the findings of this study are openly available in the National Center for Biotechnology Information (NCBI) at https://www.ncbi.nlm.nih.gov/, reference number MT246302.
